# Indoleamine 2,3-dioxygenase is a critical resistance mechanism in anti-tumor T cell immunotherapy targeting CTLA-4

**DOI:** 10.1186/2051-1426-1-S1-P77

**Published:** 2013-11-07

**Authors:** Rikke B Holmgaard, Dmitriy Zamarin, David H Munn, Jedd D Wolchok, James P Allison

**Affiliations:** 1 Department of Immunology, Memorial Sloan-Kettering Cancer Center, New York, NY, USA; 2 Department of Immunology, MD Anderson Cancer Center, Houston, TX, USA; 3 Cancer Center and Department of Pediatrics, Georgia Regents University, Augusta, GA, USA

## 

The CTLA-4 blocking antibody ipilimumab results in durable responses in metastatic melanoma, though therapeutic benefit has been limited to a fraction of patients. This calls for identification of resistance mechanisms and development of combinatorial strategies. We examine the inhibitory role of indoleamine 2,3-dioxygenase (IDO) on the anti-tumor efficacy of CTLA-4 blockade. In IDO knockout mice treated with anti-CTLA-4 antibody, we demonstrate a striking delay in B16 melanoma tumor growth and increased overall survival when compared to wild type mice. To highlight the therapeutic relevance of these findings, we show that CTLA-4 blockade strongly synergizes with IDO inhibitors to mediate rejection of both IDO-expressing and non-expressing poorly immunogenic tumors, emphasizing the importance of the inhibitory role of both tumor- and host-derived IDO. This effect was T-cell dependent, leading to enhanced infiltration of tumor-specific effector T cells and a marked increase in the effector to regulatory T cell ratios in the tumors. Overall, these data demonstrate the immunosuppressive role of IDO in the context of anti-CTLA-4 immunotherapy and provide a strong incentive to clinically explore combination therapies utilizing IDO inhibitors irrespective of IDO expression by the tumor cells.

